# Probiotics for prevention of necrotizing enterocolitis: a systematic review of current evidences

**DOI:** 10.1186/1824-7288-40-S2-A32

**Published:** 2014-10-09

**Authors:** Mario De Curtis, Lucia Dito, Francesca Conte, Gianluca Terrin

**Affiliations:** 1 Department of Pediatrics and Neuropsychiatry, University of Rome La Sapienza, Italy; 2 Department of Gynecology-Obstetrics and Perinatal Medicine, University of Rome La Sapienza, Italy

## Background

The use of probiotics has been proposed to reduce the incidence of necrotizing enterocolitis (NEC) in preterm neonates.

## Aims

To systematically review the evidences regarding the use of probiotics in preterm neonates to prevent NEC.

## Methods

We revised studies with high level of evidence (randomized clinical trials and meta-analysis). Database: MEDLINE and Pubmed. Search term: Probiotic (AND) Very Low Birth Weight (AND) necrotizing enterocolitis. Limits: Randomized clinical trial (AND) Meta-analysis.

## Results

We analyzed 20 manuscripts (13 Randomized clinical trial and 7 Meta-analysis) that were published from 2005 to 2014 and that analyze 3025 neonates. Analysis of the best evidences revealed that probiotics may have beneficial effects in the prevention of NEC, however current studies have failed to control for numerous confounding variables such as breast feeding rates, antibiotic exposure, feeding practices, and environmental cross-contamination. The incidence of NEC (stage > 2) was significantly lowered only in infants weighing 1001 to 1500 g. Thus, there is not enough evidence to support the efficacy of probiotics in extremely low birth-weight infants, and future well-designed studies are needed. Trials performed at institutions with high NEC rates have observed significant benefit from probiotics, while those institutions with low NEC rates have shown limited effects. Multistrain probiotics may be more effective than single-strain products. Currently, data from about 3000 neonates indicates that significant adverse effects of probiotics are rare.

## Conclusions

Probiotics appear to be effective in preventing NEC only in specific setting. Although reports of probiotic-related sepsis are limited, caution should be used when considering probiotic supplementation in infants at greatest risk for an impaired mucosal barrier. Policies regarding storage, preparation, distribution, administration and documentation of probiotics to ensure patient safety should be adopted.

